# A Facile Procedure for One-Pot Stable Conjugation of Two Proglucagon Cysteine-Containing Peptide Analogs

**DOI:** 10.3389/fendo.2021.693958

**Published:** 2021-08-18

**Authors:** Rongjun He, Stephanie A. Mowery, Joseph Chabenne, Brian Finan, John P. Mayer, Richard D. DiMarchi

**Affiliations:** ^1^Novo Nordisk Research Center, Indianapolis, IN, United States; ^2^Department of Molecular, Cellular & Developmental Biology, University of Colorado, Boulder, CO, United States; ^3^Department of Chemistry, Indiana University, Bloomington, IN, United States

**Keywords:** peptide, conjugation, cysteine, maleimide, thioether, glucagon, GLP-1

## Abstract

Optimization of peptides for therapeutic purposes often includes chemical conjugation or modification with substituents that serve to broaden pharmacology or improve pharmacokinetics. We report a convenient and rapid procedure for one-pot, site-specific conjugation of two cysteine-containing peptides that utilizes a bivalent linker comprising maleimide and iodoacetyl functional groups. Following maleimide-mediated peptide conjugation the linker was converted from an unstable thiosuccinimide to a stable thioether bond suitable for biological study by mild aqueous hydrolysis. The procedure is exemplified by peptide-peptide, peptide-small molecule, and peptide-fatty acid conjugations. The method provides a facile approach to search for enhanced biological outcomes through additive and sustained peptide pharmacology unencumbered by the prospect of chemical rearrangement in the course of biological study.

## Introduction

Peptide therapeutics constitute an important drug class for the treatment of multiple disease states, including cancer and diabetes (1). Chemical modifications are commonly employed to enhance the biophysical, pharmacokinetic and pharmacological properties of native peptides for optimal therapeutic performance. Prominent examples include the once-weekly GLP-1 receptor agonist semaglutide (2), the basal insulin degludec (3), and the recently approved Lutetium-DOTATATE (4). Peptide conjugation can generate single molecules capable of simultaneously targeting multiple targets to provide superior pharmacology, or an improved therapeutic index (5). The integration of two hormonal activity to single peptides is a proven concept to enhance pharmacology in metabolic diseases with several co-agonists having advanced in clinical study (6–8). A linker which joins the two molecules is an essential requirement in this type of structural optimization. Biomolecular conjugations are commonly facilitated through site specific reactions at the N-terminus or side chains, such as a lysine amine or cysteine thiol (9–11). Although many homo-bifunctional and hetero-bifunctional linkers such as DBDBP, LC-SPDP, DSS, SIAB and MBS have been reported (12), their application often requires orthogonal protection involving intermediate synthetic steps and purification. We sought a more straightforward method to achieve peptide chemical modification and heterodimerization.

We envisioned this possible through a single-pot procedure in which cysteine alone could be employed. Cysteine is an attractive candidate given its relatively low abundance in native proteins and its versatility in chemo-selective reactions, including alkylation, Michael addition, and disulfide bond formation (11). Maleimides are well-recognized to rapidly react with sulfhydryl groups under multiple reaction conditions, while a haloacetyl reacts readily with thiols under mildly alkaline conditions. Consequently, a chemical linker composed of maleimide and iodoacetyl groups might lend itself to bi-functional cysteine conjugation in a one-pot approach in similar fashion to the linker reported for protein assembly on a DNA template (13). A potential liability of the maleimido strategy is the tendency of the resultant thiosuccinimide to undergo retro-Michael β-elimination or reaction with plasma-derived thiols, most notably glutathione (14, 15). Suitable stability can be achieved through hydrolysis-based ring opening of the imide to a stable thioether bond (14, 15).

## Methods

### Peptide Synthesis

All reagents and solvents were purchased from Sigma-Aldrich (St. Louis, MO, USA), Fisher Scientific (Waltham, MA, USA) or VWR (Radnor, PA, USA) and were used without further purification. Protected amino acids and H-Rink amide ChemMatrix resin were obtained from Gyros Protein Technologies (Tucson, AZ, USA), Midwest Biotech (Fishers, IN, USA), or Chem-Impex International (Wood Dale, IL, USA). Peptide synthesis was performed either on Symphony Peptide Synthesizer or on Applied Biosystems 433A Peptide Synthesizer using preprogrammed solid-phase Fmoc protocol designed for 0.1mmol syntheses. For Applied Biosystems 433A Peptide Synthesizer, DIC/HOBt-Cl in NMP were used for coupling and 20% piperidine in NMP for deprotection. For Symphony Peptide Synthesizer, DIC/OxymaPure in DMF were used for coupling and 20% piperidine in NMP for deprotection. After assembly of the peptide sequence, the peptide bound resin was treated with cleavage cocktail (10 mL, 90% TFA, 2.5% TIS, 2.5% H2O, 2.5% 2-mercaptoethanol, 2.5% anisole) to cleave the peptide from the resin. The cleavage solution was treated with ether (30 mL) to precipitate the impure peptide, which was washed with ether another two times (30 mL each time). LC-MS analyses were performed on an Agilent 1260 Infinity/6120 Quadrupole instrument with Kinetex C8 2.6μ (75×4.6 mm, Phenomenex Inc., Torrance, CA, USA) column using a flow rate of 1 mL/min and a gradient of 20%–100% B over 10 min unless otherwise mentioned. Eluent A is water with 0.05% TFA and eluent B is 10% water, 90% MeCN, 0.05% TFA. MS collected in 200–2000 m/z range. Preparative HPLC purifications were performed on Waters instrument (Pump 2545, Detector 2489, Fraction Collector III) with Luna 10μ C8 (2) 100A AXIA (250×21.2 mm) or Kinetex 5μ C8 100A AXIA (250×21.2 mm) column at the flow rate of 12 mL/min, eluent A is 90% water, 10% MeCN, 0.1%TFA, eluent B is MeCN with 0.1% TFA. A gradient would run from 10% to 50% B in 60 minutes unless otherwise mentioned. Fractions were collected based on UV absorption at 220 nm. Cysteine-containing glucagon analog (peptide 2) and cysteine-containing GLP-1 analog were synthesized with corresponding amino acid residues in about 30% yield with >95% purity.

### Synthesis of Linker 1

To N-(2-Aminoethyl)maleimide HCl (53 mg, 0.3 mmol, 1.0 eq.) in DMF (1 mL) was added iodoacetic anhydride (116.8 mg, 0.33 mmol, 1.1 eq.) and DIEA (104.5 uL, 0.6 mmol, 2.0 eq.), and the reaction mixture was stirred at rt for 30 min. The mixture was subjected to HPLC purification (gradient 0% B to 10% B in 60 minutes), and fraction were collected and lyophilized to afford oil like product 1 (N-(2-(2,5-dioxo-2,5-dihydro-1H-pyrrol-1-yl)ethyl)-2-iodoacetamide, 14.8 mg, yield 16%). LC-MS (gradient 10% B to 80% B in 10 minutes) retention time 2.36 min, MS analyzed for C8H9IN2O3K+ 346.9, found 347.0.

### Procedure for One-Pot Heterodimerization in Liquid Phase

*Step 1:* to glucagon analog **2** (5.5 mg, 1.26 umol, 1.0 eq.) in pH 6.0 50 mM phosphate buffer (1 mL) and MeCN (0.5 mL) was added linker 1 (37.8 uL, 0.05 M in MeCN, 1.5 eq.), the mixture was stirred at rt for 30 minutes, LC-MS analysis showed the completion of Michael addition between glucagon and linker to afford intermediate peptide 3.

*Step 2:* to the reaction mixture from step 1 was added GLP-1 C-terminal cysteine analog (10.4 mg, 2.52 umol, 2.0 eq.) and pH 10.0 100 mM sodium carbonate/bicarbonate buffer (0.67 mL), the mixture was stirred at rt for 30 minutes, LC-MS analysis showed the completion of alkylation between the C-terminal cysteine GLP-1 analog and linker to afford intermediate peptide 4.

*Step 3:* to the reaction mixture from step 2 was added pH 10.0 100 mM sodium carbonate/bicarbonate buffer (0.33 mL), the mixture was measured to be pH 9.6, and it was stirred at 37°C for 1 h, LC-MS analysis showed the completion of hydrolysis of thiosuccinimide to afford final peptide conjugation product **5** in 51% isolated yield after reversed phase HPLC purification.

The conversion of peptides 6 through 11 were by the same procedure, except that peptide 6 and 7 required 3h in step 3 for hydrolysis to be completed. Peptide 6 to 11 synthesis yields were calculated based on UV absorption at 280 nm using their respective molar extinction coefficient, which was calculated from the sum of the molar extinction coefficients of individual residues with Trp (5500), Tyr (1490), and Cys (125).

### *In Vitro* Glucagon and GLP-1 Receptor Activation

Receptor activation data was collected utilizing stably transfected BHK cells over-expressing either the human GLP-1R or GcgR and firefly luciferase reporter gene linked to the cAMP response element. The cells were maintained in continuous culture in DMEM supplemented with 10% HI FBS, 300 ug/mL hygromycin, and 500 ug/mL G418 at 37°C with 5% CO_2_. Cells were plated in the supplemented DMEM at 5,000 cells per well in 96 well poly-D-Lysine coated Corning BioCoat plates and incubated overnight at 37°C with 5% CO_2._ After the overnight incubation, the media was removed from the cell plates, washed once with DPBS, and 50uL of the assay buffer (DMEM without phenol red, 10 mM Hepes, 1x Glutamax, 1% ovalbumin, and 0.1% Pluronic F-68) was added to the plate. In a separate 96 well plate, the test compounds were serially diluted by 3.5-fold across the row of the plate to create a 12-point dilution curve. Aliquots of the dilution curve were added to the cell plate in a volume of 50 uL to result in a final concentration range of the test compound from 1 × 10^-14^ to 1 × 10^-7^ M. The assay plates were incubated for 3 hours at 37°C and 5% CO_2._ After the incubation, the assay plates were washed once in DPBS. Following the wash, 100 uL of DPBS was added to each well of the plates followed by 100 uL of the steadylite plus reagent (PerkinElmer). The assay plates were covered to protect from light, incubated at room temperature with shaking at 250 rpm for 30 minutes and read using a microtiter plate reader. The data was plotted using GraphPad Prism 9.0.1 with the nonlinear regression log(agonist) *vs.* response analysis to obtain the EC_50_ for each compound. Additionally, GraphPad Prism was used to complete the statistical analysis of the EC50 values with n=6 was using the Student’s unpaired t-test analysis.

## Results and Discussion

**Scheme 1:**
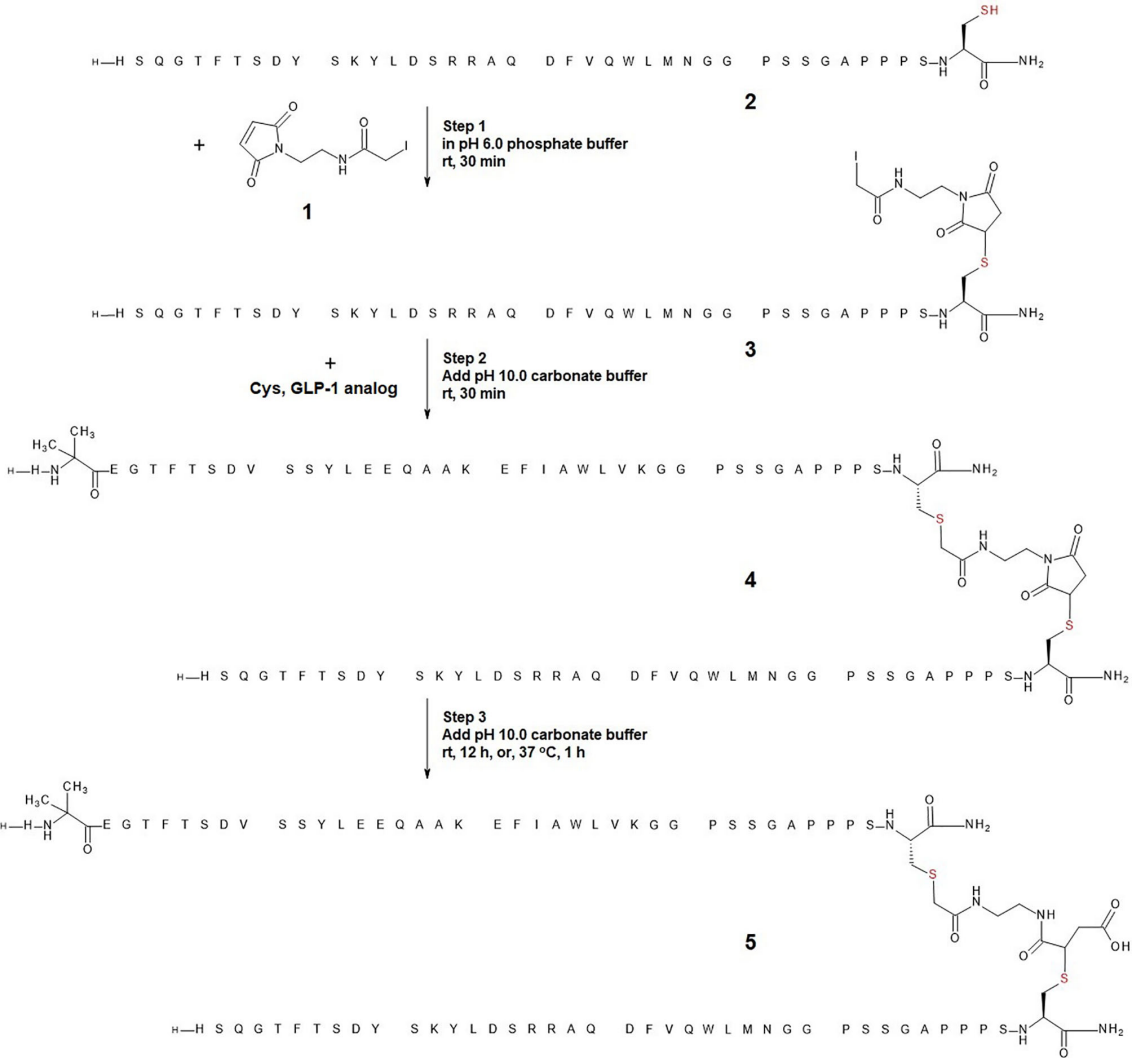
Optimized reaction conditions for one-pot heterodimerization in three steps. In step 1, glucagon analog (peptide 2) was reacted with linker 1 in pH 6.0 phosphate buffer to yield a glucagon analog-linker adduct (peptide 3). In step 2, pH 10.0 carbonate buffer was added to raise the pH of the reaction mixture to 8.3, and C-terminal extended GLP-1 cysteine analog was added to react with peptide 3 to yield a glucagon-linker-GLP-1 analog conjugate (peptide 4). In step 3, additional pH 10.0 carbonate buffer was added to further raise the pH of the reaction mixture to 9.6 such that peptide 4 was hydrolyzed to provide a chemically stable glucagon-linker(hydrolyzed)-GLP-1 analog (peptide 5).

**Figure 1:**
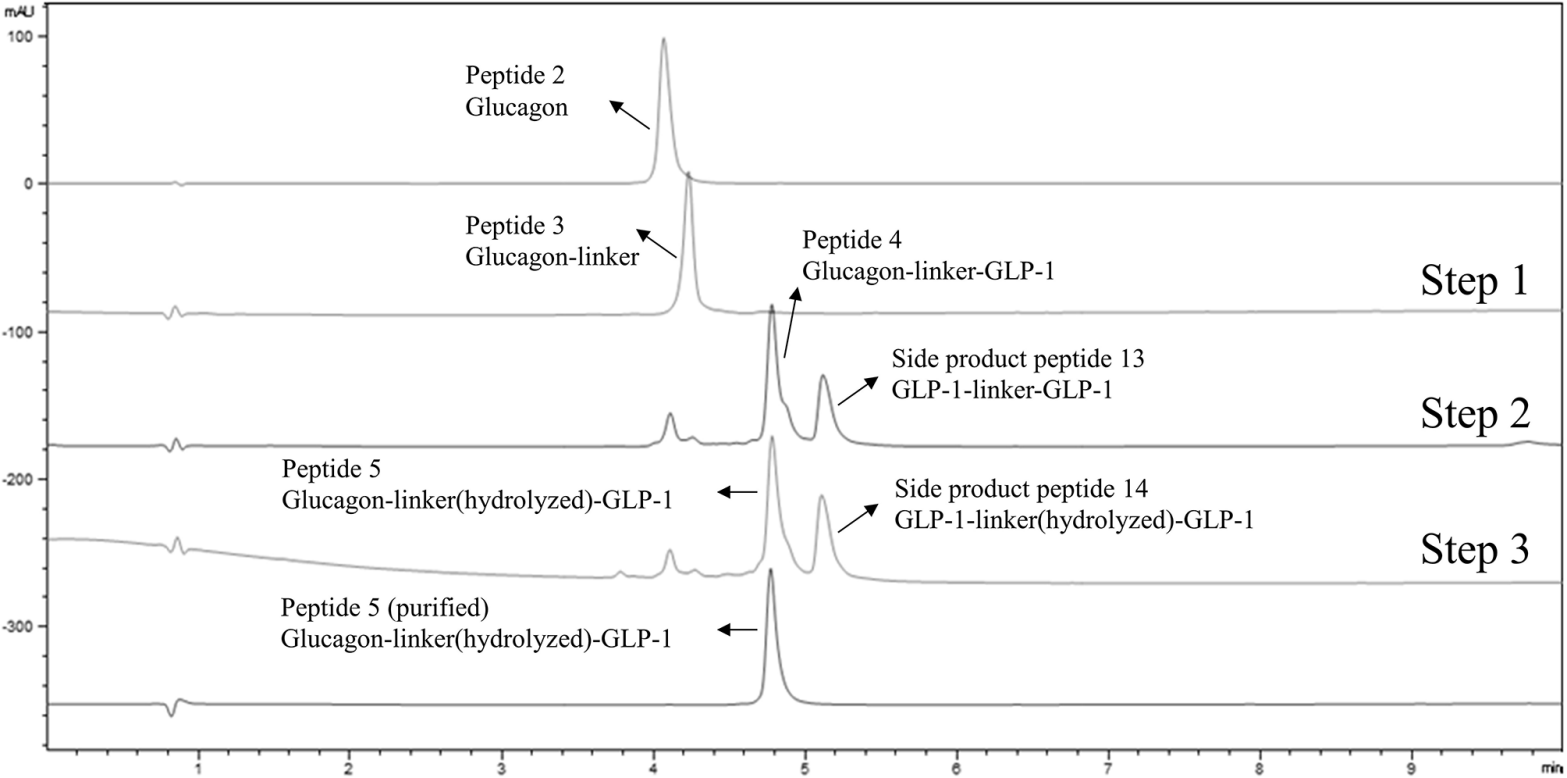
LC-MS analysis of one-pot heterodimerization in three steps. From top to bottom, the first line shows the UV absorption trace of a purified cysteine containing glucagon analog (peptide 2); the second line is the UV absorption trace of a glucagon analog-linker adduct (peptide 3) in the reaction mixture following the first step reaction; the third line shows the UV absorption trace of glucagon-linker-GLP-1 analog adduct (peptide 4) in the reaction mixture after the second step reaction, along with a minor side-product 1 representing GLP-1-linker-GLP-1 analog; the fourth line shows the UV absorption trace of the glucagon-linker(hydrolyzed)-GLP-1 adduct analog (peptide 5) in the reaction mixture following the third reaction step, along with a minor side product 2 of GLP-1-linker(hydrolyzed)-GLP-1 analog; the fifth line shows the UV absorption trace of purified peptide 5 glucagon-linker(hydrolyzed)-GLP-1 analog adduct after HPLC purification.

We employed cysteine containing glucagon and GLP-1 analogues as model peptides to test the feasibility of the envisioned one-pot conjugation using a bifunctional linker (**1,** **Scheme 1**). Cysteine was added to the C-termini of the respective peptides to facilitate tail-to-tail heterodimerization. The coupling reaction of glucagon analog bearing a C-terminal exendin-like tail (**2,** **Scheme 1**) and the linker (**1,** **Scheme 1**) was conducted in PBS buffer (pH 7.4). Analysis of the Michael addition product detected a minor side-reaction resulting from a competing alkylation. In contrast, when the reaction was conducted (in 50 mM phosphate buffer) at a slightly reduced pH of 6.0, the product was exclusively the glucagon analog-linker adduct 3 (**Scheme 1**). The linker-based iodoacetyl group proved stable under these slightly acidic reaction conditions for an extended duration. The alkylation of **3** with the C-terminally cysteine extended GLP-1 in step 2 was facilitated by increasing the pH to 8.3 (through a twofold addition of pH 10 carbonate buffer to the initial pH 6.0 maleimide reaction solution). Under these conditions the cysteine of the C-terminally extended GLP-1 analog was rapidly and fully consumed to yield the desired thiosuccinimide (**4**, **Scheme 1**). The third and final step constituted base-mediated hydrolysis and ring opening of the thiosuccinimide to provide a stable peptide heterodimer as a thioether (**5,** **Scheme 1**). We observed that prolonging alkylation time at pH 8.3 did not result in sufficient thiosuccinimide hydrolysis. Accordingly, dilution of the reaction with pH 10, carbonate buffer (equal amount relative to the initial volume of pH 6.0, phosphate buffer) was used to increase the pH to 9.6. At this pH value the hydrolysis was completed after approximately 12 hours at room temperature, or within 1 hour at 37°C without detectable degradation of the conjugate (**5,** **Scheme 1**). The final product 5 was isolated in 51% yield. LC-MS analysis of starting material (peptide 2), reaction mixtures of steps 1-3, and purified product 5 are shown in **Figure 1**.

The ability to hydrolyze the thiosuccinimide intermediate **1** to a stable thioether is greatly influenced by substituents linked to the maleimide group. The widely used maleimide-caproyl (mc) linker requires much harsher conditions for hydrolysis (45°C, pH 9.2, 48 hours) (14), which can complicate its application to macromolecules of marginal chemical stability. Alternatively, there are reports employing more labile linkers, including one that undergoes spontaneous hydrolysis at neutral pH (15). However, the more labile nature can present challenges during synthesis, where more stability may be required. In this regard, we observed that the enhanced stability of linker 1 in the pH range of 6.0-8.3 is more advantageous than that of the self-hydrolysable maleimido linker.

**Figure 2:**
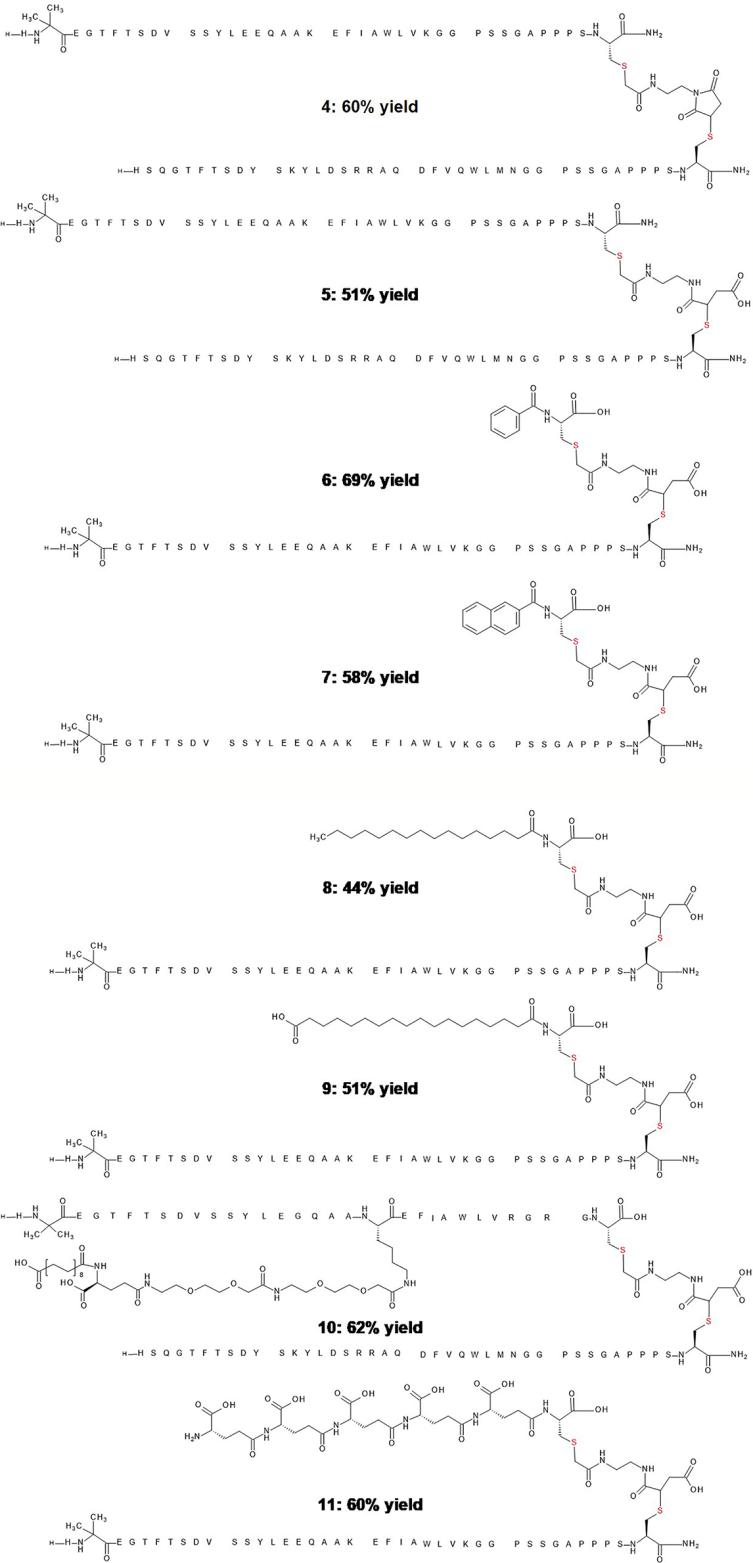
Representative examples and yields of stably conjugated peptide dimers and other chemical derivatives. Peptide 4 is the conjugate of glucagon and GLP-1 analogs before hydrolysis. Peptide 5 is the hydrolyzed product of glucagon and GLP-1 analog. Peptide 6 is the hydrolyzed product of a GLP-1 analog and benzoic acid. Peptide 7 is the hydrolyzed product of a GLP-1 analog and 2-naphthoic acid. Peptide 8 is the hydrolyzed product of a GLP-1 analog and fatty acid C16 conjugate. Peptide 9 is the hydrolyzed product of a GLP-1 analog and fatty acid C18 conjugate. Peptide 10 is the hydrolyzed product of a glucagon analog and fatty acid protracted GLP-1analog. Peptide 11 is the hydrolyzed product of a GLP-1 analog and a pentapeptide of glutamic acid.

A separate consideration for one-pot conjugation pertains to chemical modification with molecules designed to extend duration of action, or in the attachment of non-peptide pharmacological agents such as nuclear hormones (16), cytotoxic drugs (17), and oligonucleotides (18). To model the conjugation of a conventional small molecule drug for purposes of tissue targeted delivery, or multimode pharmacology we conducted a reaction of the C-terminal -extended GLP-1 cysteine analog with benzoic acid or 2-naphthoic acid *via* bivalent linker 1. The reactions proceeded rapidly, and the corresponding products were recovered in respective yields of 69% and 58% (**Figure 2**, peptide 6 and 7). We anticipate that this and related linkers can be used to conjugate peptides and proteins with other various molecular entities, such as fluorophores for molecular imaging, antigens for antibody generation or recognition, and oligonucleotides for selective gene therapy.

Modification of peptides with long chain fatty acids has emerged as a well-validated strategy to enhance pharmacokinetic properties, minimize proteolysis, slow renal clearance, and alter biodistribution (19). Frequently, the fatty acids are introduced in the course of solid-phase peptide synthesis employing an orthogonal protection scheme. Site-specific conjugation in solution is more challenging given the presence of competing functionalities. Accordingly, we explored the use of the cysteine thiol group to site-specifically conjugate fatty acids such as C16, C18 diacid and simplify such peptide modification. The modified fatty acids were conjugated to GLP-1 related peptides bearing an exendin-like peptide extension with a single C-terminal cysteine in 44% to 51% respective yields using this simplified conjugation approach (**Figure 2**, peptide 8 and 9). In the case of the fatty acylated conjugates the hydrolysis in step 3 required a slightly longer reaction for completion (3 h at 37°C), likely the result of the increased hydrophobicity adjacent to the thiosuccinimide.

To examine the conjugation reaction efficiency in the presence of a fatty acid, we added a cysteine residue to the C-terminus of semaglutide. This GLP-1 agonist is a C18 diacid protracted GLP-1 analogue which allows once weekly dosing for the treatment of type 2 diabetes (2). The C18 diacid in semaglutide did not appear to noticeably impact conjugation efficiency as the conjugated and hydrolyzed product was recovered in 62% yield (**Figure 2**, peptide 10), under conditions identical to those used for the prior GLP-1 peptide analogs without side-chain fatty acylation. Aqueous solubility and stability are frequently encountered problems in the optimization of peptide therapeutic candidates. A popular approach to circumvent this challenge is to alter the isoelectric point of the peptide. One such example involves the addition of multiple glutamic acid residues to the side chain of a specific residue by stepwise synthesis (20). Linker **1** was used in a one pot fashion to conjugate multiple glutamic acids to an analogue of GLP-1 in respectable yield (**Figure 2**, peptide 11).

**Figure 3:**
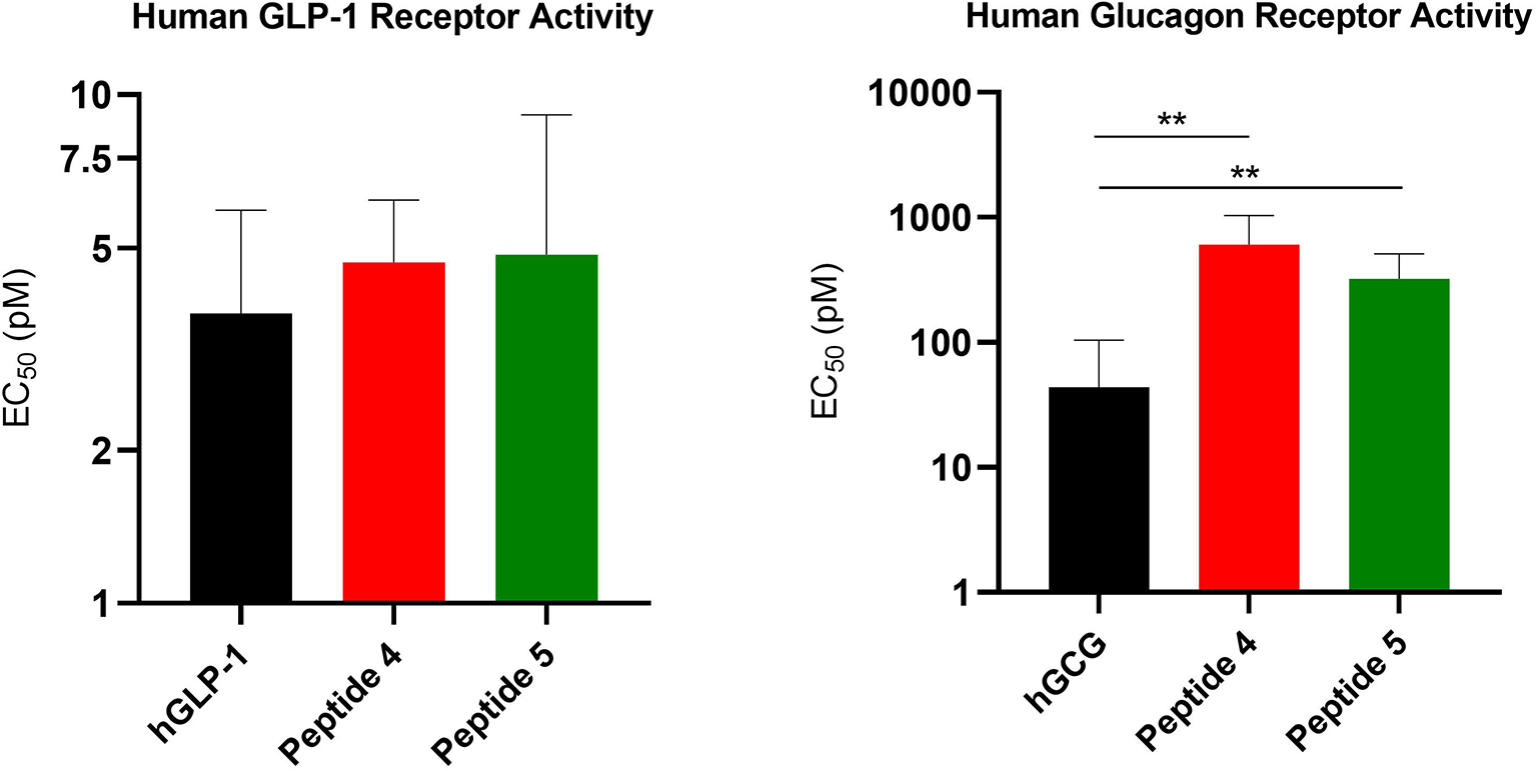
The *in vitro* bioactivity assessment of peptides 4 and 5. Assays were conducted with BHK cells that over-express either the human GLP-1 or glucagon receptors. Left panel, human GLP-1 receptor where native GLP-1 has a determined EC_50_ of 3.72 +/- 2.21 pM (n=6), and peptides 4 and 5 are 4.68 +/- 1.53 pM (n=6) and 4.85 +/- 4.29 pM (n=6), respectively (no statistical significance between human GLP-1, peptide 4, and peptide 5, using the Student’s unpaired t-test analysis). Right panel, human glucagon receptor where native glucagon has an EC_50_ of 43.8 +/- 60.41pM (n=6) and peptides 4 and 5 are 607.1 +/- 428.3 pM (n=6) and 323.0 +/- 186.6 pM (n=6), respectively (P < 0.01 between hGCG and peptide 4, P < 0.01 between hGCG and peptide 5, no statistical significance between peptide 4 and peptide 5, using the Student’s unpaired t-test analysis). **P < 0.01.

To validate the biological integrity of a conjugated product following hydrolysis at pH 9.6, we assessed *in vitro* bioactivity of the conjugated peptide 4 and the corresponding hydrolyzed analogue 5, at human GLP-1 and glucagon receptors (21). Peptides 4 and 5 demonstrated comparable potency to native GLP-1 in stimulating cAMP concentrations at GLP-1 receptors (**Figure 3**). Native human GLP-1 displayed an EC_50_ of 3.72 +/- 2.21 pM in human GLP-1 receptor, peptides **4** and **5** were measured to be of similarly high potency with respective EC_50_ values of 4.68 +/- 1.53 pM and 4.85 +/- 4.29 pM (no statistical significance between human GLP-1, peptide 4, and peptide 5, using the Student’s unpaired t-test analysis). Native human glucagon displayed an EC_50_ of 43.8 +/- 60.41pM at human GCG receptor, peptide **4** and **5** showed reduced but comparable potency with respective EC_50_ values of 607.1 +/- 428.3 pM and 323.0 +/- 186.6 pM from each other (P < 0.01 between hGCG and peptide 4, P < 0.01 between hGCG and peptide 5, no statistical significance between peptide 4 and peptide 5, using the Student’s unpaired t-test analysis). The reduced glucagon potency reflects the specific site selection. If a more balanced potency is the objective a positional scan with spacer optimization is a required next step, but in this specific co-agonist the glucagon activity is typically targeted for much less relative activity comparable to what is reported here to minimize the potential for hyperglycemia (8, 22).

## Conclusion

Use of a bifunctional linker described in this report offers a straightforward and rapid route to the synthesis of chemically modified peptides under aqueous conditions. This approach takes advantage of differential, pH dependent reactivity of maleimide and iodoacetyl functional groups. The maleimide reacted exclusively with cysteine at pH 6.0, while the iodoacetyl group reacted with the cysteine at pH 8.3. Importantly, the linker-derived thiosuccinimide underwent facile hydrolysis and ring opening to yield a stable thioether under mild aqueous conditions. The single-pot, three step procedure afforded conjugated products in moderate to good yields, and representative conjugates retained full receptor agonism in which the relative potency was a function of site selection in conjugation. We have chosen to employ peptide analogs of glucagon and GLP-1 as models where the C-terminus has been extended with an exendin-like peptide sequence including a single terminal cysteine. Such modification of glucagon has dramatically enhanced the aqueous solubility in physiological buffers and provides a molecular spacer for conjugation purposes (23). The impact of this C-terminal extension on GLP-1 physical properties was first observed by Eng and associates in exendin-based peptides (24), and further assessed later in GLP-1 antagonists (25). The magnitude of relative activity in each peptide component is something that will need to be explored and in certain instances optimized to varying degrees with each individual peptide. Peptides 4 and 5 are heterodimers of glucagon and GLP-1 analogs that demonstrate much enhanced GLP-1 receptor activity relative to glucagon, which has been a medicinal objective (8). Chemical modification with lipids and large molecule pegylation at residues 10 or 24 of glucagon has demonstrated retention of greater activity (21, 26).

The inherent virtue in this approach is the potential for rapid profiling of combinatorial mixtures to explore the prospect for complementary and synergistic activities that can sizably improve biological outcomes when two biological mechanisms are integrated as a single molecular entity. The combination of biological activity derived from individual receptors within the proglucagon family and related receptors has previously demonstrated significant increase in peptide potency and total efficacy (7, 21). Should such molecular pairs be identified there are a host of customary medicinal chemistry methods that can be assessed for further maturation aligned with drug development objectives. However, the number of potential pairings given the vast library of peptide candidates, some without apparent individual efficacy and others such as orphan ligands without any known biological function places a premium on a rapid method to screen a broad field. Therein resides the attraction of a facile method to rapidly detect the more promising pairs to which precise chemical optimization can be subsequently employed to prioritize those combinations that demonstrate transformative biological responses relative to pharmacology with individual peptides, or physical mixtures.

## Data Availability Statement

The original contributions presented in the study are included in the article/**Supplementary Material**. Further inquiries can be directed to the corresponding author.

## Author Contributions

RH and RD planned and designed the study. RH, SM, and JC performed experiments. RH, SM, JC, JM, BF performed data analysis and interpretation. BF, RD supervised the study. All authors contributed to the article and approved the submitted version.

## Conflict of Interest

Authors RH, SM, JC, and BF were employed by Novo Nordisk.

The remaining authors declare that the research was conducted in the absence of any commercial or financial relationships that could be construed as a potential conflict of interest.

## Publisher’s Note

All claims expressed in this article are solely those of the authors and do not necessarily represent those of their affiliated organizations, or those of the publisher, the editors and the reviewers. Any product that may be evaluated in this article, or claim that may be made by its manufacturer, is not guaranteed or endorsed by the publisher.
