# Supplementary figures and images for: A Facile Procedure for One-Pot Stable Conjugation of Two Proglucagon Cysteine-Containing Peptide Analogs
Source: Front Endocrinol (Lausanne). 2021 Aug 18;12:693958. doi: 10.3389/fendo.2021.693958 (PMC8416343; doi:10.3389/fendo.2021.693958)

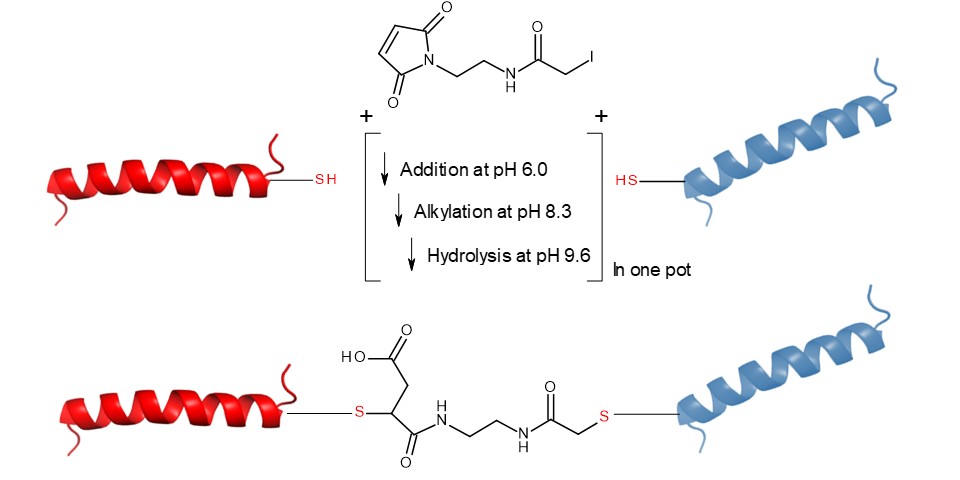

Supplement: Supplementary file 1 [file Image_1.jpeg]
