# Supplementary material for: A Facile Procedure for One-Pot Stable Conjugation of Two Proglucagon Cysteine-Containing Peptide Analogs
Source: Front Endocrinol (Lausanne). 2021 Aug 18;12:693958. doi: 10.3389/fendo.2021.693958 (PMC8416343; doi:10.3389/fendo.2021.693958)
Supplement: Supplementary file 2 [file Table_1.docx]

A Facile Procedure for One-Pot Stable Conjugation of Two Cysteine Containing Peptides

**Rongjun He^1^, Stephanie A. Mowery^1^, Joseph Chabenne^1^, Brian Finan^1^, John P. Mayer^2^, Richard D. DiMarchi^3*^**

^1^Novo Nordisk Research Center, Indianapolis, IN 46241, USA

^2^Department of Molecular, Developmental & Cell Biology, University of Colorado, Boulder, CO, 80309, USA

^3^Department of Chemistry, Indiana University, Bloomington, IN 47405, USA

*** Correspondence:**Corresponding Author
rdimarch@indiana.edu

**Table of Contents**

| **Content** | **Page number** |
| --- | --- |
| LC-MS spectra of linker 1 | 2 |
| 1H and 13C NMR spectra of linker 1 | 3 |
| LC-MS spectra of glucagon peptide **2** | 4 |
| LC-MS spectra of GLP-1 peptide **12** | 5 |
| LC-MS spectra of intermediate peptide **3** in reaction mixture of step 1 | 6 |
| LC-MS spectra of intermediate peptide **4** (retention time 4.8 min) and side intermediate peptide **13** (retention time 5.1 min) in reaction mixture of step 2 | 7-9 |
| LC-MS spectra of hydrolyzed peptide conjugation product **5** (retention time 4.8 min) and side intermediate peptide **14** (retention time 5.1 min) in reaction mixture of step 3 | 9-10 |
| LC-MS spectra of reaction of peptide conjugation product **6** (retention time 4.8 min) | 11 |
| LC-MS spectra of reaction of peptide conjugation product **7** (retention time 5.0 min) | 12 |
| LC-MS spectra of reaction of peptide conjugation product **8** (retention time 6.3 min) | 13 |
| LC-MS spectra of reaction of peptide conjugation product **9** (retention time 5.7 min) | 14 |
| LC-MS spectra of reaction of peptide conjugation product **10** (retention time 5.3 min) | 15 |
| LC-MS spectra of reaction of peptide conjugation product **11** (retention time 5.0 min) | 16 |

**LC-MS spectra of linker 1**


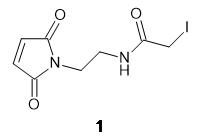

**1H and 13C NMR spectra of linker 1**


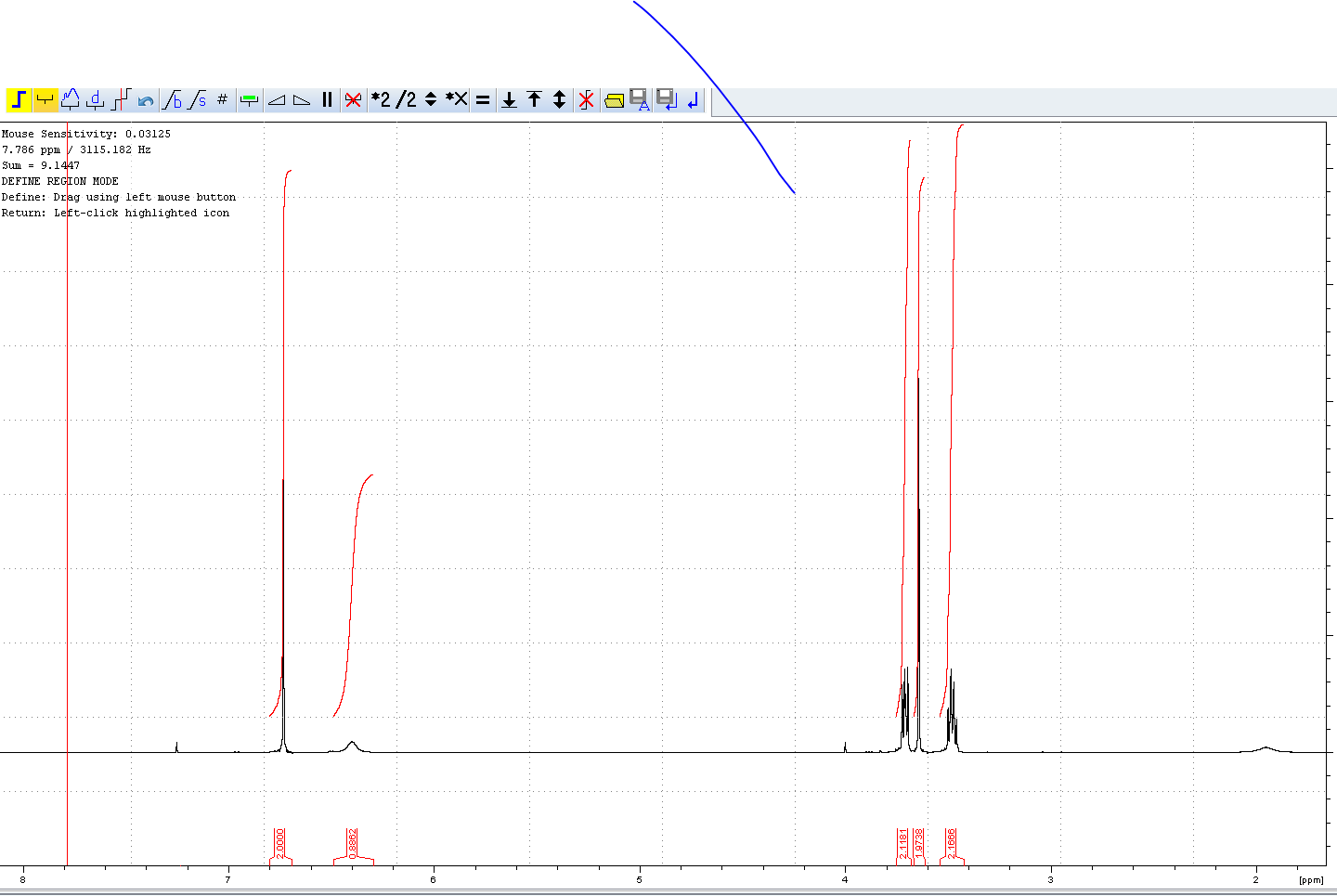


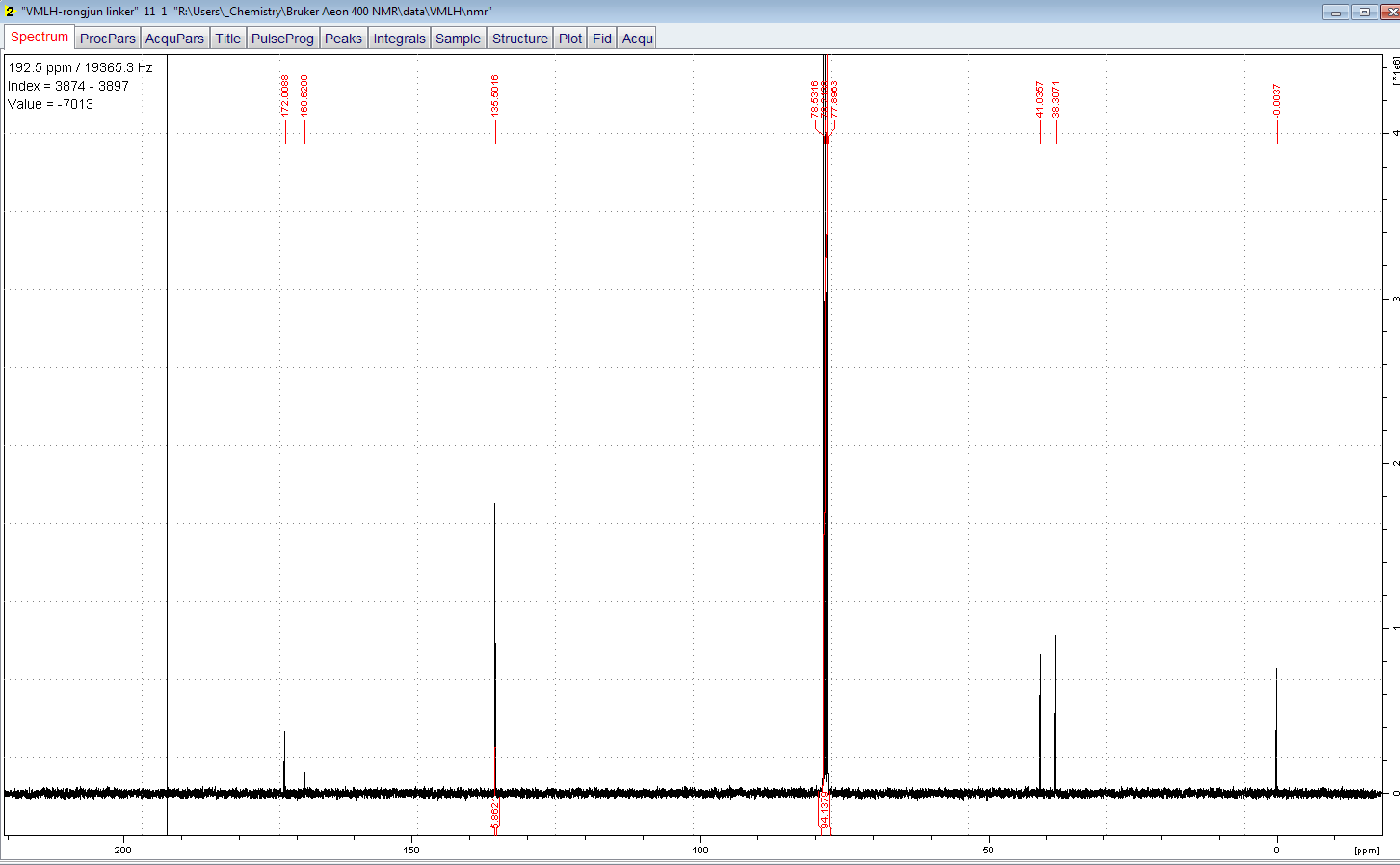


**LC-MS spectra of glucagon peptide 2**


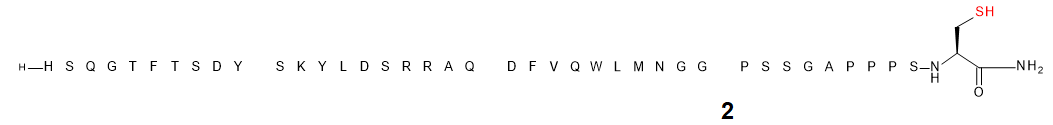


| Ave Mass: | 4375.73 |  | (M+5H)5+ | 876.146 |
| --- | --- | --- | --- | --- |
| (M+H)+: | 4376.73797 |  | (M+6H)6+ | 730.288333 |
| (M+2H)2+ | 2188.87297 |  | (M+7H)7+ | 626.104286 |
| (M+3H)3+ | 1459.58464 |  | (M+8H)8+ | 547.96625 |
| (M+4H)4+ | 1094.94047 |  | (M+9H)9+ | 487.192222 |

**LC-MS spectra of GLP-1 peptide 12**


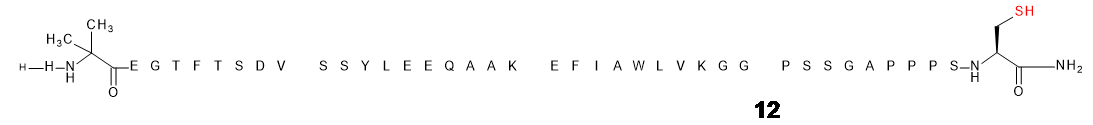


| Ave Mass: | 4165.55 |  | (M+5H)5+ | 834.11 |
| --- | --- | --- | --- | --- |
| (M+H)+: | 4166.55797 |  | (M+6H)6+ | 695.258333 |
| (M+2H)2+ | 2083.78297 |  | (M+7H)7+ | 596.078571 |
| (M+3H)3+ | 1389.52464 |  | (M+8H)8+ | 521.69375 |
| (M+4H)4+ | 1042.39547 |  | (M+9H)9+ | 463.838889 |

**LC-MS spectra of intermediate peptide 3 in reaction mixture of step 1**


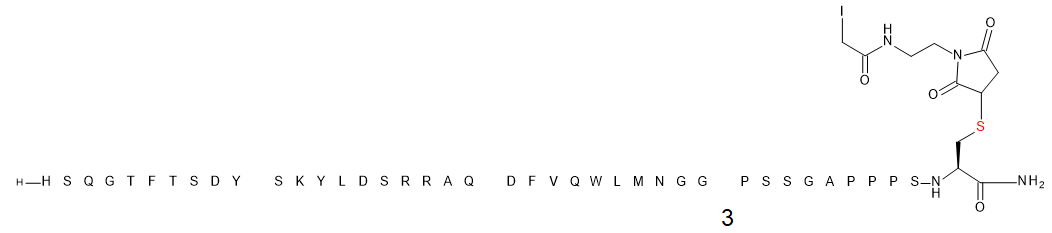


| Ave Mass: | 4683.8 |  | (M+5H)5+ | 937.76 |
| --- | --- | --- | --- | --- |
| (M+H)+: | 4684.80797 |  | (M+6H)6+ | 781.633333 |
| (M+2H)2+ | 2342.90797 |  | (M+7H)7+ | 670.114286 |
| (M+3H)3+ | 1562.27464 |  | (M+8H)8+ | 586.475 |
| (M+4H)4+ | 1171.95797 |  | (M+9H)9+ | 521.422222 |

**LC-MS spectra of intermediate peptide 4 (retention time 4.8 min) and side intermediate peptide 13 (retention time 5.1 min) in reaction mixture of step 2**


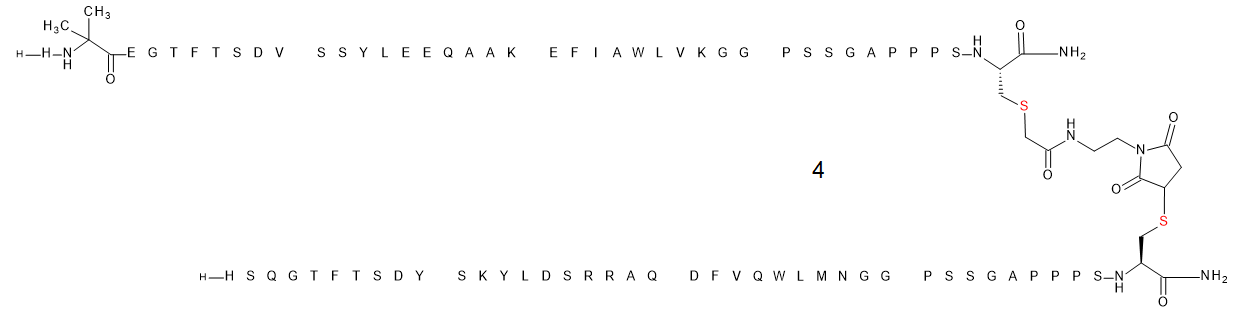


| Ave Mass: | 8721.44 |  | (M+5H)5+ | 1745.288 |
| --- | --- | --- | --- | --- |
| (M+H)+: | 8722.44797 |  | (M+6H)6+ | 1454.57333 |
| (M+2H)2+ | 4361.72797 |  | (M+7H)7+ | 1246.92 |
| (M+3H)3+ | 2908.15464 |  | (M+8H)8+ | 1091.18 |
| (M+4H)4+ | 2181.36797 |  | (M+9H)9+ | 970.048889 |


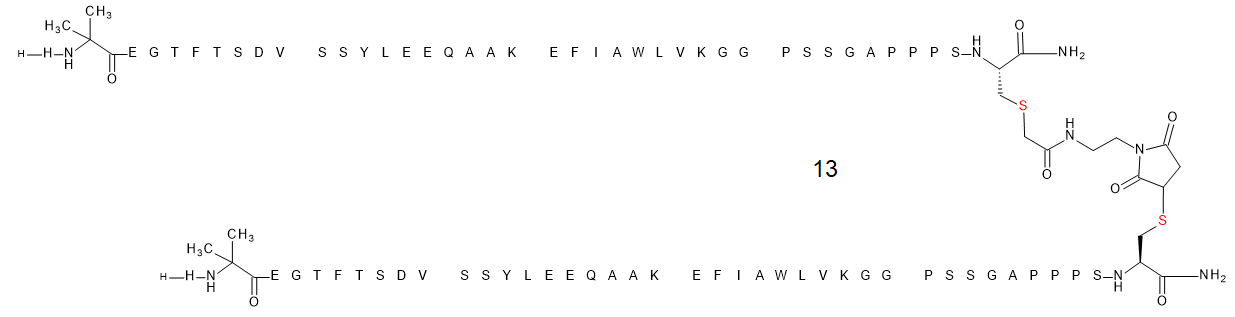


| Ave Mass: | 8511.2 |  | (M+5H)5+ | 1703.24 |
| --- | --- | --- | --- | --- |
| (M+H)+: | 8512.20797 |  | (M+6H)6+ | 1419.53333 |
| (M+2H)2+ | 4256.60797 |  | (M+7H)7+ | 1216.88571 |
| (M+3H)3+ | 2838.07464 |  | (M+8H)8+ | 1064.9 |
| (M+4H)4+ | 2128.80797 |  | (M+9H)9+ | 946.688889 |

**LC-MS spectra of hydrolyzed peptide conjugation product 5 (retention time 4.8 min) and side intermediate peptide 14 (retention time 5.1 min) in reaction mixture of step 3**


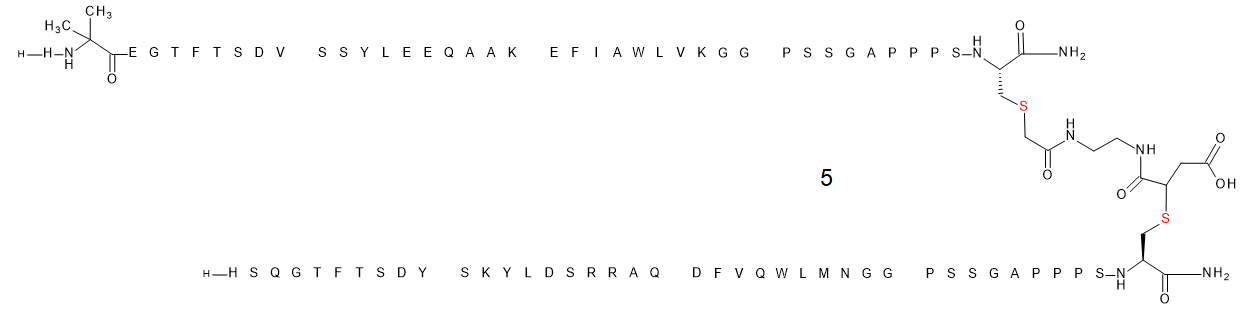


| Ave Mass: | 8739.45 |  | (M+5H)5+ | 1748.89 |
| --- | --- | --- | --- | --- |
| (M+H)+: | 8740.45797 |  | (M+6H)6+ | 1457.575 |
| (M+2H)2+ | 4370.73297 |  | (M+7H)7+ | 1249.49286 |
| (M+3H)3+ | 2914.15797 |  | (M+8H)8+ | 1093.43125 |
| (M+4H)4+ | 2185.87047 |  | (M+9H)9+ | 972.05 |


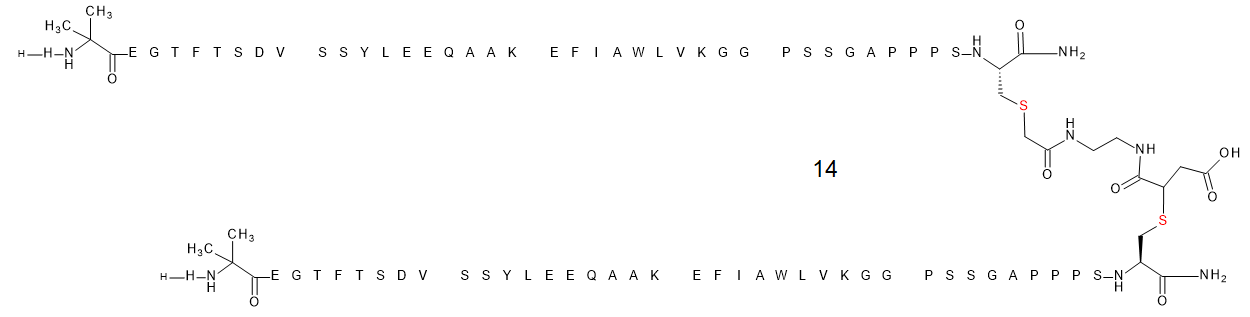


| Ave Mass: | 8529.3 |  | (M+5H)5+ | 1706.86 |
| --- | --- | --- | --- | --- |
| (M+H)+: | 8530.30797 |  | (M+6H)6+ | 1422.55 |
| (M+2H)2+ | 4265.65797 |  | (M+7H)7+ | 1219.47143 |
| (M+3H)3+ | 2844.10797 |  | (M+8H)8+ | 1067.1625 |
| (M+4H)4+ | 2133.33297 |  | (M+9H)9+ | 948.7 |

**LC-MS spectra of reaction of peptide conjugation product 6 (retention time 4.8 min)**


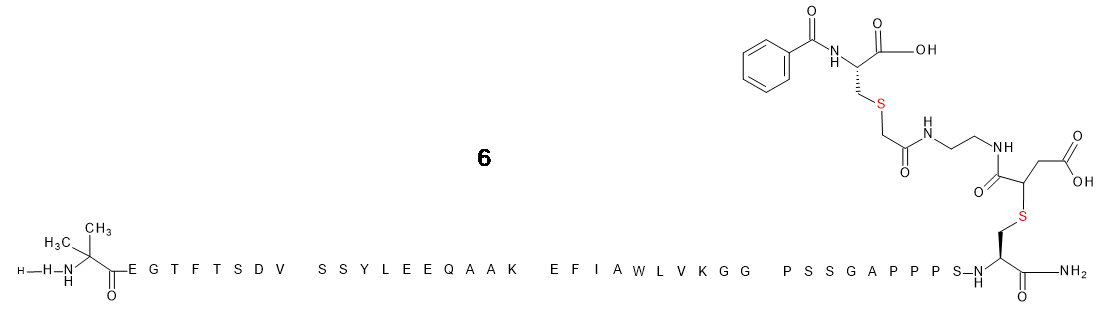


| Ave Mass: | 4588.99 |  | (M+5H)5+ | 918.798 |
| --- | --- | --- | --- | --- |
| (M+H)+: | 4589.99797 |  | (M+6H)6+ | 765.831667 |
| (M+2H)2+ | 2295.50297 |  | (M+7H)7+ | 656.57 |
| (M+3H)3+ | 1530.6713 |  | (M+8H)8+ | 574.62375 |
| (M+4H)4+ | 1148.25547 |  | (M+9H)9+ | 510.887778 |

**LC-MS spectra of reaction of peptide conjugation product 7 (retention time 5.0 min)**


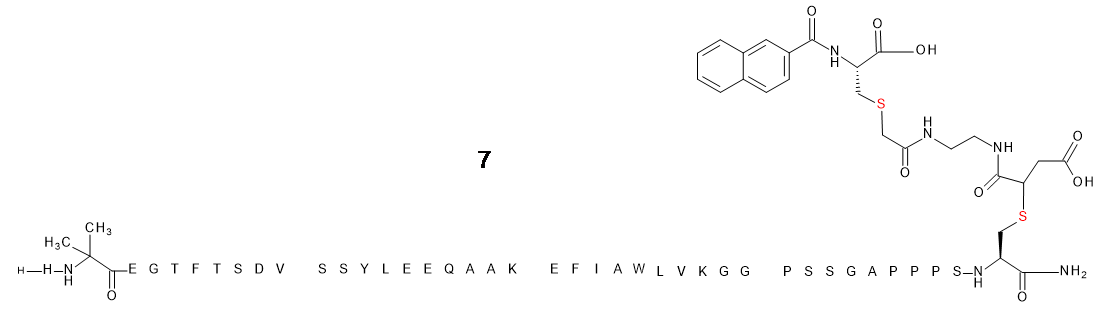


| Ave Mass: | 4639.05 |  | (M+5H)5+ | 928.81 |
| --- | --- | --- | --- | --- |
| (M+H)+: | 4640.05797 |  | (M+6H)6+ | 774.175 |
| (M+2H)2+ | 2320.53297 |  | (M+7H)7+ | 663.721429 |
| (M+3H)3+ | 1547.35797 |  | (M+8H)8+ | 580.88125 |
| (M+4H)4+ | 1160.77047 |  | (M+9H)9+ | 516.45 |

**LC-MS spectra of reaction of peptide conjugation product 8 (retention time 6.3 min)**


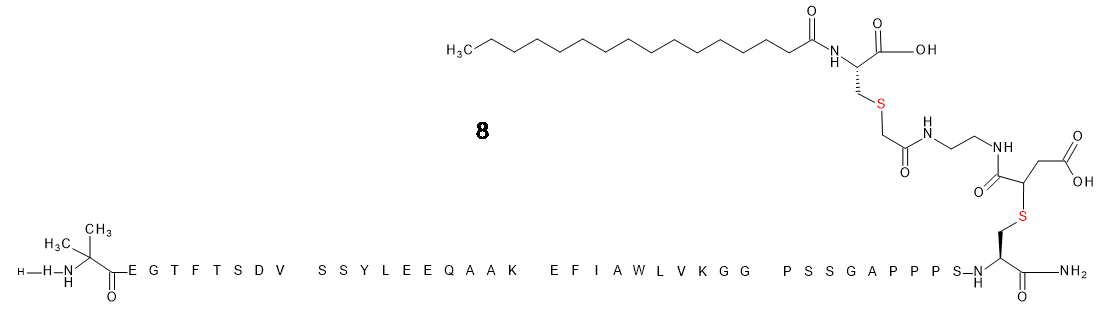


| Ave Mass: | 4723.29 |  | (M+5H)5+ | 945.658 |
| --- | --- | --- | --- | --- |
| (M+H)+: | 4724.29797 |  | (M+6H)6+ | 788.215 |
| (M+2H)2+ | 2362.65297 |  | (M+7H)7+ | 675.755714 |
| (M+3H)3+ | 1575.43797 |  | (M+8H)8+ | 591.41125 |
| (M+4H)4+ | 1181.83047 |  | (M+9H)9+ | 525.81 |

**LC-MS spectra of reaction of peptide conjugation product 9 (retention time 5.7 min)**


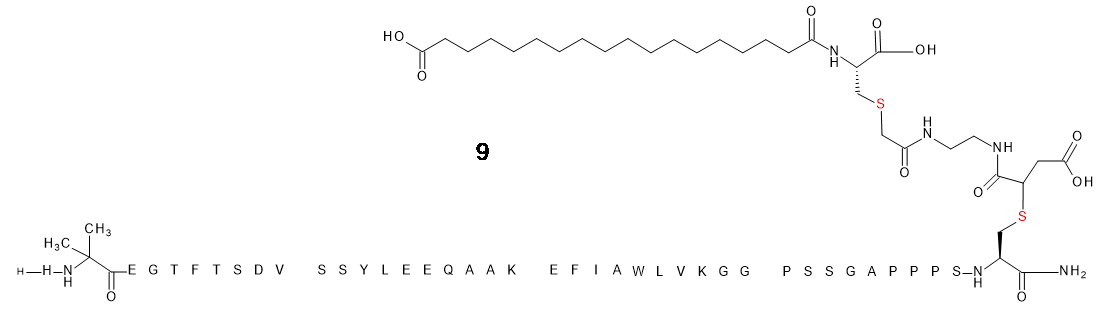


| Ave Mass: | 4781.33 |  | (M+5H)5+ | 957.266 |
| --- | --- | --- | --- | --- |
| (M+H)+: | 4782.33797 |  | (M+6H)6+ | 797.888333 |
| (M+2H)2+ | 2391.67297 |  | (M+7H)7+ | 684.047143 |
| (M+3H)3+ | 1594.78464 |  | (M+8H)8+ | 598.66625 |
| (M+4H)4+ | 1196.34047 |  | (M+9H)9+ | 532.258889 |

**LC-MS spectra of reaction of product 10 (retention time 5.3 min)**


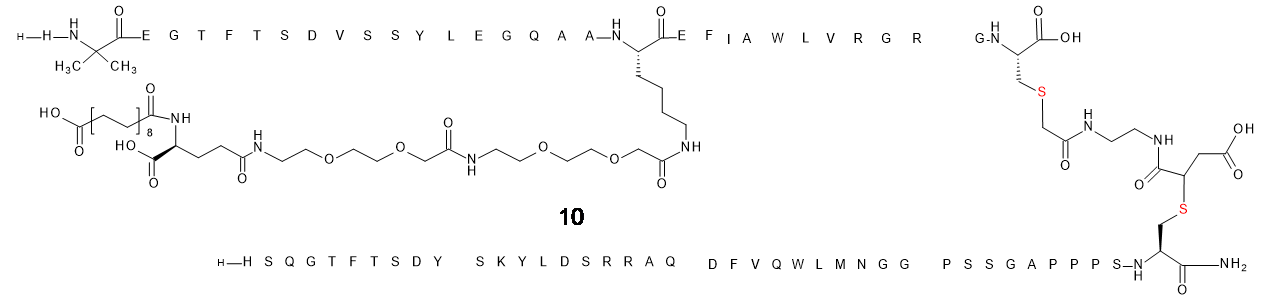


| Ave Mass: | 8790.62 |  | (M+5H)5+ | 1759.124 |
| --- | --- | --- | --- | --- |
| (M+H)+: | 8791.62797 |  | (M+6H)6+ | 1466.10333 |
| (M+2H)2+ | 4396.31797 |  | (M+7H)7+ | 1256.80286 |
| (M+3H)3+ | 2931.21464 |  | (M+8H)8+ | 1099.8275 |
| (M+4H)4+ | 2198.66297 |  | (M+9H)9+ | 977.735556 |

**LC-MS spectra of reaction of peptide conjugation product 11 (retention time 5.0 min)**


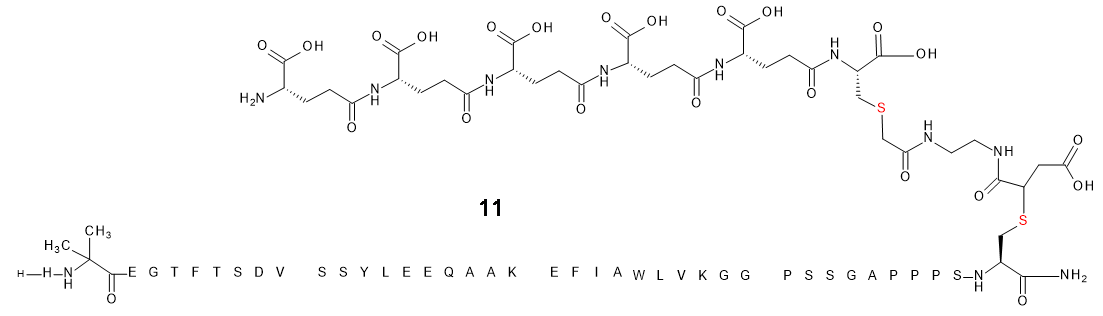


| Ave Mass: | 5130.45 |  | (M+5H)5+ | 1027.09 |
| --- | --- | --- | --- | --- |
| (M+H)+: | 5131.45797 |  | (M+6H)6+ | 856.075 |
| (M+2H)2+ | 2566.23297 |  | (M+7H)7+ | 733.921429 |
| (M+3H)3+ | 1711.15797 |  | (M+8H)8+ | 642.30625 |
| (M+4H)4+ | 1283.62047 |  | (M+9H)9+ | 571.05 |
